# [Retracted] AKR1C1 alleviates LPS‑induced ALI in mice by activating the JAK2/STAT3 signaling pathway

**DOI:** 10.3892/mmr.2025.13449

**Published:** 2025-01-29

**Authors:** Xianjun Wang, Baocheng Yang, Yuyu Li, Jiye Luo, Yanli Wang

Mol Med Rep 24: 833, 2021; DOI: 10.3892/mmr.2021.12473

Following the publication of this paper, it was drawn to the Editor's attention by a concerned reader that certain of the immunohistochemical data shown in Fig. 1C on p. 5 were strikingly similar to data appearing in different form in another article written by different authors at different research institutes that had already been published in the journal *Archives of Biochemistry and Biophysics* prior to the submission of this paper to *Molecular Medicine Reports*. In view of the fact that the abovementioned data had already apparently been published previously, the Editor of *Molecular Medicine Reports* has decided that this paper should be retracted from the Journal. After having been in contact with the authors, they accepted the decision to retract the paper. The Editor apologizes to the readership for any inconvenience caused.

